# Fine mapping of the major bleomycin-induced pulmonary fibrosis susceptibility locus in mice

**DOI:** 10.1007/s00335-018-9774-3

**Published:** 2018-09-01

**Authors:** Marie-Eve Bergeron, Anguel Stefanov, Christina K. Haston

**Affiliations:** 1 Meakins-Christie Laboratories McGill University, Montreal, PQ Canada; 2 Present Address: 2Department of Mathematics, Statistics, Physics, and Computer Science, I.K. Barber School of Arts and Sciences, The University of British Columbia | Okanagan, ASC 347 - 3187 University Way, Kelowna, BC V1V 1V7 Canada

## Abstract

Susceptibility to fibrotic lung disease differs among people and among inbred strains of mice exposed to bleomycin where C57BL/6J mice are susceptible and C3H/HeJ mice are spared fibrotic disease. Genetic mapping studies completed in offspring derived from these inbred strains revealed the inheritance of C57BL/6J alleles at loci, including the major locus on chromosome 17, called *Blmpf1* bleomycin-induced pulmonary fibrosis 1, to be linked to pulmonary fibrosis in treated mice. In the present study, to reduce the interval of *Blmpf1*, we bred and phenotyped a panel of subcongenic mice with C3H/HeJ alleles in a C57BL/6J background. Subcongenic mice received bleomycin via osmotic minipump and the fibrosis phenotype was measured histologically. Inheritance of C3H/HeJ alleles from 34.31 to 35.02 Mb was revealed to spare bleomycin-induced pulmonary fibrosis of C57BL/6J mice. From database analysis, 40 protein coding genes have been mapped to this reduced *Blmpf1* interval, 18 of which contain C57BL/6J:C3H/HeJ sequence polymorphisms predicted to affect protein structure or to confer allele-dependent expression, and by RT-PCR analysis of lung tissue, we show 6 of these genes to differ in expression between C57BL/6J and C3H/HeJ mice. Genes known to regulate T cell numbers and activation (*Btnl* family, *Notch4*) are among the limited list of potential causal variants leading to lung disease in this model and the bronchoalveolar lavage of protected subcongenic mice had fewer lymphocytes, post bleomycin, than did C57BL/6J mice. We conclude that *Blmpf1*genes contributing to the susceptibility to bleomycin-induced pulmonary fibrosis could alter the adaptive immune response of C57BL/6J mice.

**Electronic supplementary material:**

The online version of this article (10.1007/s00335-018-9774-3) contains supplementary material, which is available to authorized users.

## Introduction

Pulmonary fibrosis is a genetically complex disease which can result from known environmental or therapeutic exposures, or can occur idiopathically (Lederer and Martinez 2018). The pathology of excessive deposition of extracellular matrix in the lung interstitium can result in impaired lung function and, ultimately, respiratory failure. Pulmonary fibrosis is a chronic disease in which an excessive repair response to injury, characterized by fibroblast proliferation and extracellular matrix deposition, leads to compromised lung function (Lederer and Martinez 2018). This devastating disease has a prevalence of 10–60 cases per 100,000 members of the general population and a high mortality rate (Lederer and Martinez 2018). The mechanisms through which pulmonary fibrosis develops are incompletely understood but likely involve dysregulated repair of alveolar epithelial cell injury (King et al. 2011; Lederer and Martinez 2018) which may be influenced by a tissue inflammatory response (Lederer and Martinez 2018; Wynn 2011). Recent mechanistic data also point to regulation of the adaptive immune response as an important contributor to pulmonary fibrosis development (Huang et al. 2015; Kass et al. 2012; Lo Re et al. 2013; Wilson et al. 2010; Wynn 2011).

Due to its genetic and pathological complexity studies of mouse models which present clinical features of the disease can be used to uncover heritable variation predisposing to the lung response. Experimentally, bleomycin challenge has been used extensively in mice as a model of pulmonary fibrosis (Lee et al. 2014; Noble et al. 2012). Specifically, the lung phenotype of C57BL/6J mice, following a 7-day subcutaneous dose of bleomycin, consists of an alveolar inflammatory cell infiltrate with subpleural regions of fibrosis; a pathology that has been described for clinical cases of idiopathic pulmonary fibrosis (Gross and Hunninghake 2001; Lederer and Martinez 2018; Nuovo et al. 2012). This bleomycin delivery method, developed by Harrison and Lazo (1987), and used by us (Haston et al. 2005; Honeyman et al. 2013; Lemay and Haston 2005; Paun et al. 2013) has also been found to produce more fibrosis in the lung, and a fibrotic phenotype more closely resembling idiopathic pulmonary fibrosis, than the more commonly used experimental method of intratracheal drug delivery (Aono et al. 2012; Gabazza et al. 2002; Lee et al. 2014). In addition, the altered immune response evident in pulmonary fibrosis clinical cases can also be observed in bleomycin-treated C57BL/6J mice, including an increase in bronchoalveolar lavage CD4+ and CD8+ T cells (Zhu et al. 1996). T lymphocytes have been pathologically implicated in fibrosis in this model as antibody depletion of CD3+ cells has been shown to abrogate the development of the disease (Huaux et al. 2003; Sharma et al. 1996), while T regulatory cell expansion exacerbates fibrosis (Birjandi et al. 2016).

In contrast to the pulmonary fibrosis phenotype presented by C57BL/6J mice, the A/J and C3Hf/KAM or C3H/HeJ mouse strains have been shown to be spared fibrosis following bleomycin treatment (Haston et al. 2005; Lemay and Haston 2005; Paun et al. 2013), and informative genetic crosses (Haston et al. 2002; Paun et al. 2013) have mapped the major locus of susceptibility to pulmonary fibrosis to a chromosome 17 interval named bleomycin-induced pulmonary fibrosis 1 (*Blmpf1*). The existence of this fibrosis susceptibility locus has been further verified through studies of consomic B6.17^A/J^ mice, which were protected from the fibrosis phenotype (Paun et al. 2013). *Blmpf1* includes the gene-rich major histocompatibility complex, a region that has been both associated with the pulmonary fibrosis phenotype clinically (Aquino-Galvez et al. 2009; Falfán-Valencia et al. 2005; Fingerlin et al. 2016), and which harbors genes known to regulate the adaptive immune response (Rhodes et al. 2016).

Genomic investigations of gene expression profiling and genome wide association have also been completed to address the genetic basis of fibrosis susceptibility in this model. In detail, we used gene expression profiling to define strain-dependent pulmonary gene expression levels in C57BL/6J, C3H, and A/J mice (Haston et al. 2005; Lemay and Haston 2005), and pathway analyses revealed the biological processes of apoptosis and immune regulation to be significantly represented in the differential response. In addition, phenotyping of the lung injury induced by bleomycin treatment of 23 inbred strains followed by genome wide analyses was used to identify variants associated with fibrotic lung disease, including within genes mapping to *Blmpf1* (Paun et al. 2013).

Herein, we used the strategy of generating and phenotyping subcongenic mice, which carry locus-specific chromosome 17 C3H alleles in the C57BL/6J background, to reduce the linkage interval defining *Blmpf1*. Secondly, we evaluated the pulmonary expression and DNA sequence variation of the set of positional candidate genes to isolate the potential causal genetic variants influencing predisposition to pulmonary fibrosis in this model.

## Methods

### Mice and subcongenic line development

**Fig. 1:**
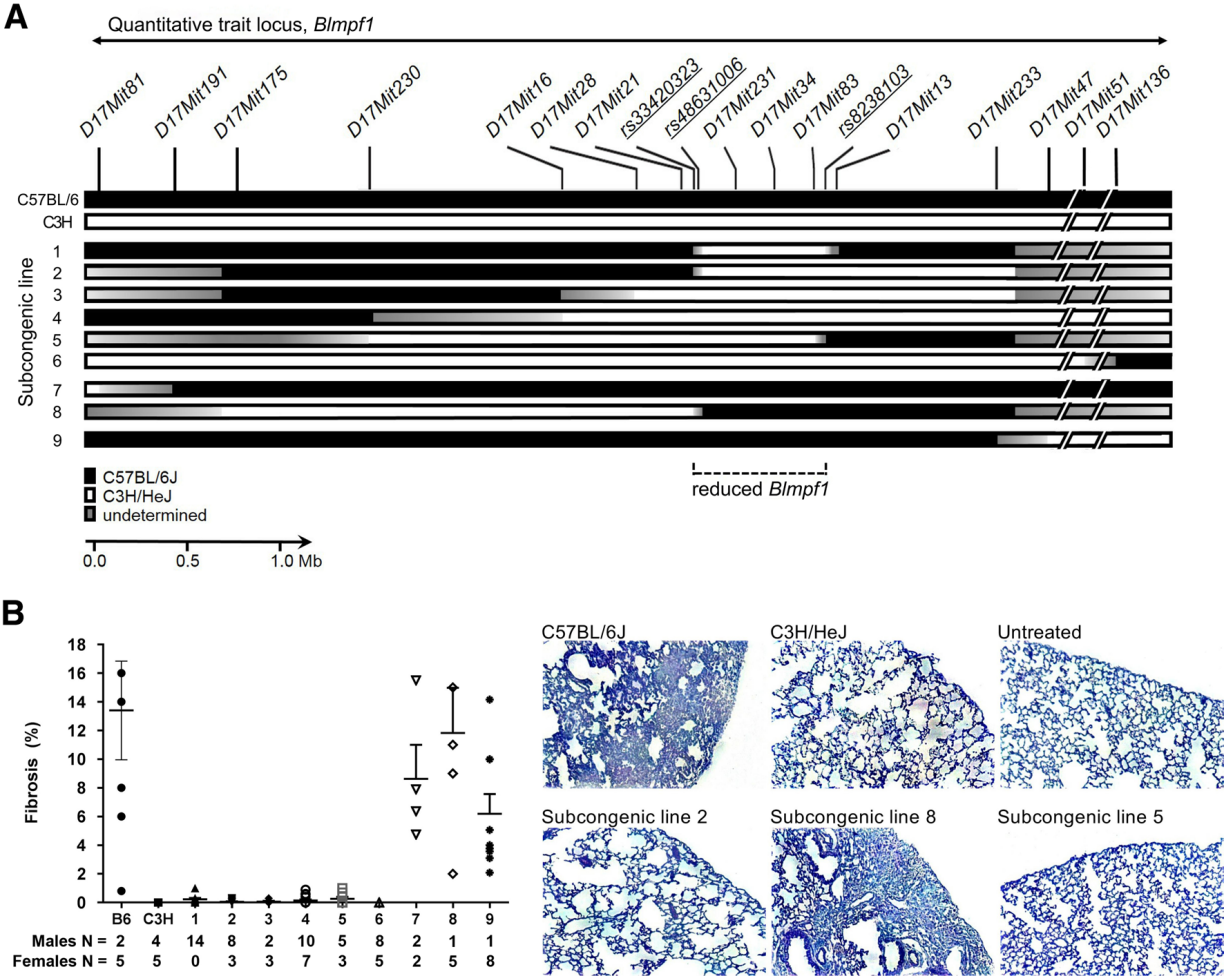
Genotype and bleomycin-induced lung phenotype of C57BL/6J, C3H/HeJ, and *Blmpf1* subcongenic mice. **a** Genotypes [C3H/HeJ alleles (white box); C57BL/6J alleles (black box); undetermined (gray box)] were assessed with microsatellite and SNP markers. **b** Mice were treated with bleomycin by osmotic minipump and euthanized 42 days later. The percentage of the lung with fibrosis was measured from image analysis of histological sections and the mean ± SEM of 4–17 bleomycin-treated mice for each subcongenic line, as indicated below figure, and for the parental strains, is given. Representative lung sections stained with Masson’s trichrome; magnification ×50. No fibrosis was detected in untreated control animals

C57BL/6J and C3H/HeJ mice were purchased from the Jackson Laboratory (Bar Harbor, ME) and housed in the animal facility of the Meakins-Christie Laboratories. From these strains, C57BL/6J:C3H/HeJF1mice were bred and were backcrossed to C57BL/6J mice. DNA was extracted from a tail piece of each resultant offspring mouse. Mice were genotyped with microsatellite markers (Dietrich et al. 1994) using primer pairs identified from the Mouse Genome Database (Smith et al. 2018). Offspring revealed to be heterozygous at the locus of interest on chromosome 17, as shown in Fig. 1, were identified and again backcrossed to C57BL/6J mice. The process was repeated for six generations and at this time, sibling pairs identified as heterozygous at the same loci were intercrossed to generate homozygous subcongenic strains. The genotypes of mice of subcongenic lines 1, 2, 5, and 8 were refined by sequencing of PCR-amplified regions containing SNPs. Positions of SNPs were taken from the UCSC genome browser GRCm38/mm10 (genome.ucsc.edu).

### Bleomycin treatment

Lung damage was elicited by administering bleomycin through osmotic minipumps implanted subcutaneously in 8–10 weeks old animals, as described previously (Harrison and Lazo 1987; Haston et al. 1996, 2005; Honeyman et al. 2013; Lemay and Haston 2005; Paun et al. 2013). As in past studies, and due to a sex difference in pulmonary fibrosis susceptibility, male mice received 100 U bleomycin/kg body weight (approximately 2.5 U/mouse), and female mice received 125 U bleomycin/kg body weight and were euthanized at 6 weeks following treatment. Untreated control mice were euthanized at the 6-week time point.

### Pulmonary fibrosis phenotyping

At the time of sacrifice, the lungs were removed and the single left lobe was perfused with 10% buffered formalin and processed histologically. Sections of lung tissue (5 µm) were stained with Masson’s Trichrome and the fibrosis score was calculated as the percentage of lung surface covered by fibrosis relative to the total lung surface (Image Pro Plus; Haston et al. 1996; Puthawala et al. 2008). Fibrosis scoring was completed by a user who was blinded to the mouse genotype and treatment.

### Candidate gene identification

Protein coding genes mapping to the confirmed minimal subcongenic interval on chromosome 17 were identified using Mouse Genome Informatics, (Genome Reference Consortium Mouse Build 38: GRCm38), verified using Ensembl, (http://www.ensembl.org) and were considered positional candidate genes for the trait of bleomycin-induced fibrosis. The positional candidate genes were assessed for the presence of allelic differences between C57BL/6J and C3H/HeJ strains which could alter the amino acid sequence (i.e., coding non-synonymous changes, essential splice site changes, premature stops), using data in Sanger release 1505 (http://www.sanger.ac.uk/sanger/Mouse_SnpViewer/rel-1505).

Secondly, the positional candidate genes with SNPs in potential regulatory regions (5′ or 3′ UTR, nonsense mediated decay) were filtered for those with strain-dependent pulmonary differential expression, in the untreated condition or post-bleomycin, using data from our gene expression profiles (Haston et al. 2005) and obtained by quantitative RT-PCR.

Finally, the positional candidate genes were assessed for their association to pulmonary fibrosis susceptibility in a panel of inbred mouse strains of known fibrosis response to bleomycin (Paun et al. 2013).

### Quantitative real-time PCR

Following sacrifice, right lungs were immediately homogenized in 2 mL of Trizol reagent and placed in dry ice. The homogenates were stored at − 85 °C until RNA isolation was completed following the manufacturer’s (Sigma, Oakville, Ontario, Canada) instructions. These experiments were performed as described previously (Haston et al. 2005; Honeyman et al. 2013; Lemay and Haston 2005). Briefly, 4–5 µg of total RNA from the right mouse lung was reverse transcribed with oligo(dT)_12−18_ Primer using Superscript™ II RNase H Reverse Transcriptase (Invitrogen, Carlsbad, CA) to make cDNA. Quantitative RT-PCR assays were completed using the Applied Biosystems International Prism 7500 Sequence Detection System and TaqMan™ assays on demand and with the reference gene Ataxin 10 (*Atxn10*, Assay Mm00450332_m1).

### Bronchoalveolar lavage fluid analysis

At the time of sacrifice, bronchoalveolar lavage collection was performed by cannulating the trachea and instilling the lungs with 1 mL of phosphate buffered saline (PBS). The lavage volume recovered from each animal was recorded and the number of cells counted using a hemocytometer. For each lavage sample, macrophages, lymphocytes, and polymorphonuclear cells were morphologically identified (×400 magnification) following hematoxylin-eosin staining (Hema-3 Staining System, Fisher Diagnostics) and are reported as a percentage of 500 counted cells.

### Data analysis

The reduced *Blmpf1* congenic interval was isolated using the sequential method (Shao et al. 2010) wherein the phenotype of the line of subcongenic mice with the minimal donor region was compared to that of the line with the next minimal region, and this comparison repeated in turn to remaining lines, to identify the minimal C3H donor region harboring the *Blmpf1* locus. Phenotypic differences between groups were evaluated using two-sided T tests performed using Microsoft Excel software. The effect sizes of the contributions of the loci, revealed in subcongenic mice, to pulmonary fibrosis susceptibility, were estimated with Cohen’s *d*.

Gene expression data were analyzed as in (Fox et al. 2013; Honeyman et al. 2013). In these analyses, the expression of each gene, relative to that of the reference gene, was determined and mean delta *Ct* values among groups compared using an analysis of variance followed by Tukey’s post tests. Differences in lavage cell differential among groups were evaluated using an analysis of variance followed by Tukey’s post tests, which were performed using GraphPad software.

## Results

### Fine mapping of Blmpf1

Following bleomycin treatment, C57BL/6J mice develop pulmonary fibrosis (Harrison and Lazo 1987) and C3H/HeJ mice do not (*P* = 2.06 × 10^− 4^) as shown in Fig. 1 and in agreement with our prior study (Haston et al. 2005). Based on data from a C57BL/6J x C3H/fKAM F2 intercross (Haston et al. 2002), *Blmpf1* extends from *D17Mit198* (27.66 Mbp in Ensembl) to *D17Mit35* (45.54 Mbp in Ensembl) for a region of 17.88 Mbp. To investigate whether C3H/HeJ alleles in specific subcongenic regions of *Blmpf1* (illustrated in Fig. 1a) could alter the bleomycin-induced pulmonary fibrosis of C57BL/6J mice, lines of subcongenic mice were bred, treated with bleomycin and their lung responses assayed histologically. A substantial reduction in the size of *Blmpf1* was achieved, as shown in Fig. 1. Specifically, mice of subcongenic line 1 contain the minimal C3H donor region, among evaluated subcongenic lines, and these mice were protected from bleomycin-induced pulmonary fibrosis, (*P* = 2.2 × 10^− 4^ vs. C57BL/6J mice) indicating that at least one *Blmpf* quantitative trait locus maps to this region. Sequential analyses of the subcongenic lines 2, 3, 4, 5, and 6, in turn, revealed no significant difference (*P* > 0.09) in the pulmonary fibrosis phenotype of mice as the C3H donor interval increased in size, thus the region in subcongenic line 1 is the minimal C3H region containing at least one *Blmpf* quantitative trait locus. In a second sequential analysis, the fibrosis phenotype of subcongenic line 7 mice did not differ from that of subcongenic line 8 mice (*P* = 0.44), or in turn from that of C57BL/6J mice (*P* = 0.85), thus the C3H donor alleles of subcongenic lines 7 and 8 do not influence bleomycin-induced pulmonary fibrosis. The phenotypes of subcongenic line 7 and 8 mice do, however, support the localization of the susceptibility locus evident from the response of subcongenic line 1 mice. Overall, the fibrosis susceptibility of the mice of subcongenic line 8 defines the proximal marker of region to be *rs33420323* (UCSC map position 34 309 602), while the resistant phenotype of line 5 indicates the distal marker to be *rs8238103* (UCSC map position 35 020 634), for an interval of 0.71 Mb. The reduced region of *Blmpf1* is also evident by analysis of data from the set of female mice alone (data not shown).

Subcongenic line 1 mice, which were resistant to fibrosis development, have the minimal C3H subcongenic region, among subcongenic lines, which wholly contains the reduced *Blmpf1*. As shown in Fig. 1 the C3H subcongenic region of this line is defined by the same proximal marker as the reduced *Blmpf1*, and the distal marker is *rs29528640* (UCSC map position 35 027 478), for an interval 6884 bp larger than reduced *Blmpf1*. Based on the phenotype of subcongenic line 1 mice, the effect size of this reduced *Blmpf1* locus is estimated to be 4.16.

Finally, the reduced fibrosis phenotype of subcongenic line 9 mice, versus that of C57BL/6J mice (*P* = 0.01), suggests a second locus in the distal portion of chr 17 may influence fibrosis susceptibility. As this region was evaluated in mice of one subcongenic line only, and in the less fibrosis-sensitive female mice, the existence of this locus requires verification. Indeed, using the data from female mice only, the fibrosis phenotype of subcongenic line 9 mice does not differ (*P* = 0.09) from that of C57BL/6J mice. The effect size of this putative locus is estimated to be 1.52.

### Reduced *Blmpf1* candidate gene evaluation

By phenotyping lines of *Blmpf1* subcongenic mice for their fibrotic responses to bleomycin, we have reduced this linkage interval to 0.71 Mb. To identify the genetic variation which potentially influences bleomycin-induced pulmonary fibrosis, we assessed each of the genes mapping to the confirmed and reduced *Blmpf1* linkage interval, the set of positional candidate genes, for inbred strain DNA sequence variation and for expression in lung tissue.

Ensembl and the Mouse Genome Informatics databases report 40 protein coding genes in this reduced region of *Blmpf1*, and using the Sanger inbred strain sequence variation data, 17 of these genes, listed in Table 1, contain C57BL/6J:C3H/HeJ DNA variants which are predicted to affect the encoded protein (missense, stop, or splice site changes).

**Table 1:** DNA sequence analysis of reduced *Blmpf1* candidate genes

Gene name	Symbol	Missense, stop or splice variants^a^	Nonsense mediated decay or 3′, 5′ variant^b^	Upstream or downstream variant^c^	Association^d^
Histocompatibility 2, class II antigen E beta2	*H2-Eb2*	4	11	40	0.65
Butyrophilin-like 2	*Btnl2*	6	15	94	0.85
Butyrophilin-like 1	*Btnl1*	2	0	18	0.23
Butyrophilin-like 4	*Btnl4*	57	0	299	0.12
Butyrophilin-like 6	*Btnl6*	89	0	185	0.004
Notch 4	*Notch4*	8	153	6	0.002
Tenascin XB	*Tnxb*	29	1	51	0.02
cDNA sequence BC051142	*BC051142*	2	1	80	0.0003
Pre B cell leukemia homeobox 2	*Pbx2*	0	2	0	No SNP
Advanced glycosylation end product-specific receptor	*Ager*	1	1	1	No SNP
FK506 binding protein-like	*Fkbpl*	1	0	1	0.23
Activating transcription factor 6 beta	*Atf6b*	1	2	12	0.04
Complement component 4B	*C4b*	6	3	46	No SNP
Complement component 4A	*C4a*	3	0	54	No SNP
Serine/threonine kinase 19	*Stk19*	2	36	36	No SNP
Ring finger protein 5	*Rnf5*	1	0	0	No SNP
Complement factor B	*Cfb*	1	0	1	0.43
Cytochrome P450, family 21, subfamily a, polypeptide 1	*Cyp21a1*	12	3	113	No SNP

^abc^Number of C3H/HeJ:C57BL/6J DNA sequence variants as categorized in Sanger database

^d^Association—minimal *P* value of association to pulmonary fibrosis in 23 inbred strains (Paun et al. 2013)

**Fig. 2:**
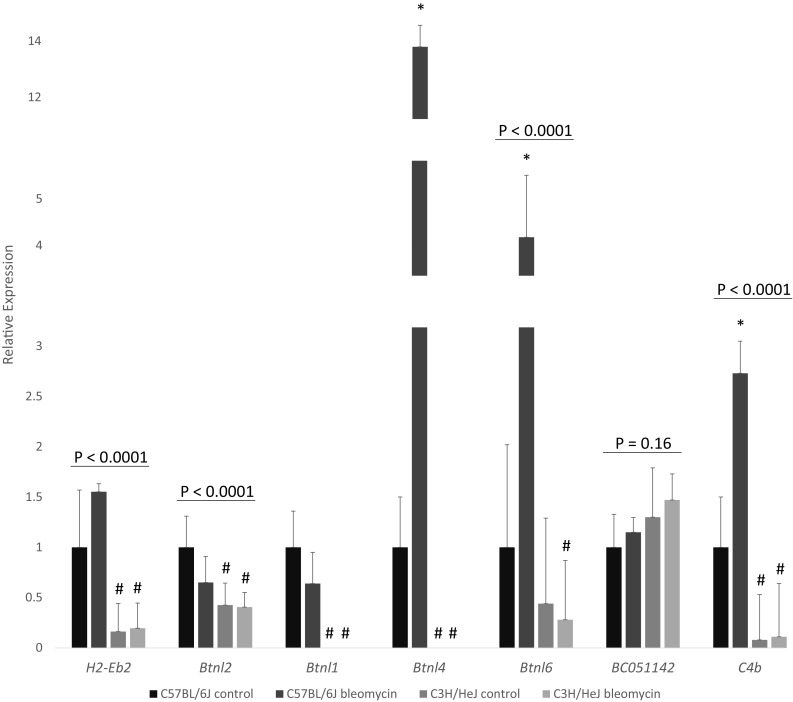
Pulmonary expression of candidate genes in the reduced *Blmpf1* region. Mice were treated as in Fig. 1 or left untreated (controls). Gene expression in right lung tissue is relative to the reference gene *Atxn10*, and normalized to C57BL/6J control level. Mean ± coefficient of variation of 6–15 mice per group. *P* values by ANOVA. Asterisk denotes a significant difference in expression of bleomycin-treated mice relative to untreated in strain controls, *P* < 0.05; hash indicates a significant difference in expression by strain, *P* < 0.05, by Tukey’s post hoc test. *Btnl1* and *Btnl4* were not detected in lungs of C3H/HeJ mice after 40 qRT-PCR cycles

To determine which of the positional candidate genes are differentially expressed by strain, in untreated mice or post bleomycin, we reviewed gene expression data from a prior experiment (Haston et al. 2005) and measured expression by RT-PCR. The pulmonary expression of 30 of the positional candidate genes had been queried by microarray (Supplemental Figure 1) and, of these, 6 genes were identified to have strain-dependent expression. We evaluated the expression levels of 7 of the remaining 10 positional candidate genes (3 predicted genes were excluded) in the lungs of C57BL/6J and C3H/HeJ mice both in the untreated condition and after bleomycin challenge. As shown in Fig. 2, 6 genes were identified to have strain-dependent pulmonary expression, including the genes *Btnl1* and *Btnl4*, for which expression was detected in the lungs of the fibrosis prone C57BL/6J mice only. A review of data from Sanger indicated 6 (listed in Table 1), of the 12 differentially expressed genes, to have C57BL/6J:C3H/HeJ DNA variants which could contribute to strain-dependent expression.

In total, 18 genes in the reduced *Blmpf1* region were identified by sequence and expression analyses to have genetic variation which potentially influences bleomycin-induced lung disease. A review of data from our study of genome wide association to fibrotic lung disease susceptibility (Paun et al. 2013) revealed 5 of the 18 genes to be significantly associated with this trait, as detailed in Table 1.

### Bleomycin-induced bronchoalveolar lavage lung phenotype

MouseSNP, association, and gene expression analysis of the positional candidate genes mapping to the reduced *Blmpf1* region point to specific genes known to regulate T cell differentiation and activation, specifically genes of the butyrophilin-like, *Btnl*, family, *Notch 4*, and *H2-Eb2*, (Amsen et al. 2015; Arnett and Viney 2014; Monzón-Casanova et al. 2016; Rhodes et al. 2016; Xia et al. 2018) as among the potential causal variants leading to fibrosis in mice. To investigate whether lymphocytes are a component of the strain-dependent lung response to bleomycin, we treated C57BL/6J mice, and mice of subcongenic line 1, incorporating the reduced *Blmpf1* region, with bleomycin and evaluated the cells of the bronchoalveolar lavage.

**Fig. 3:**
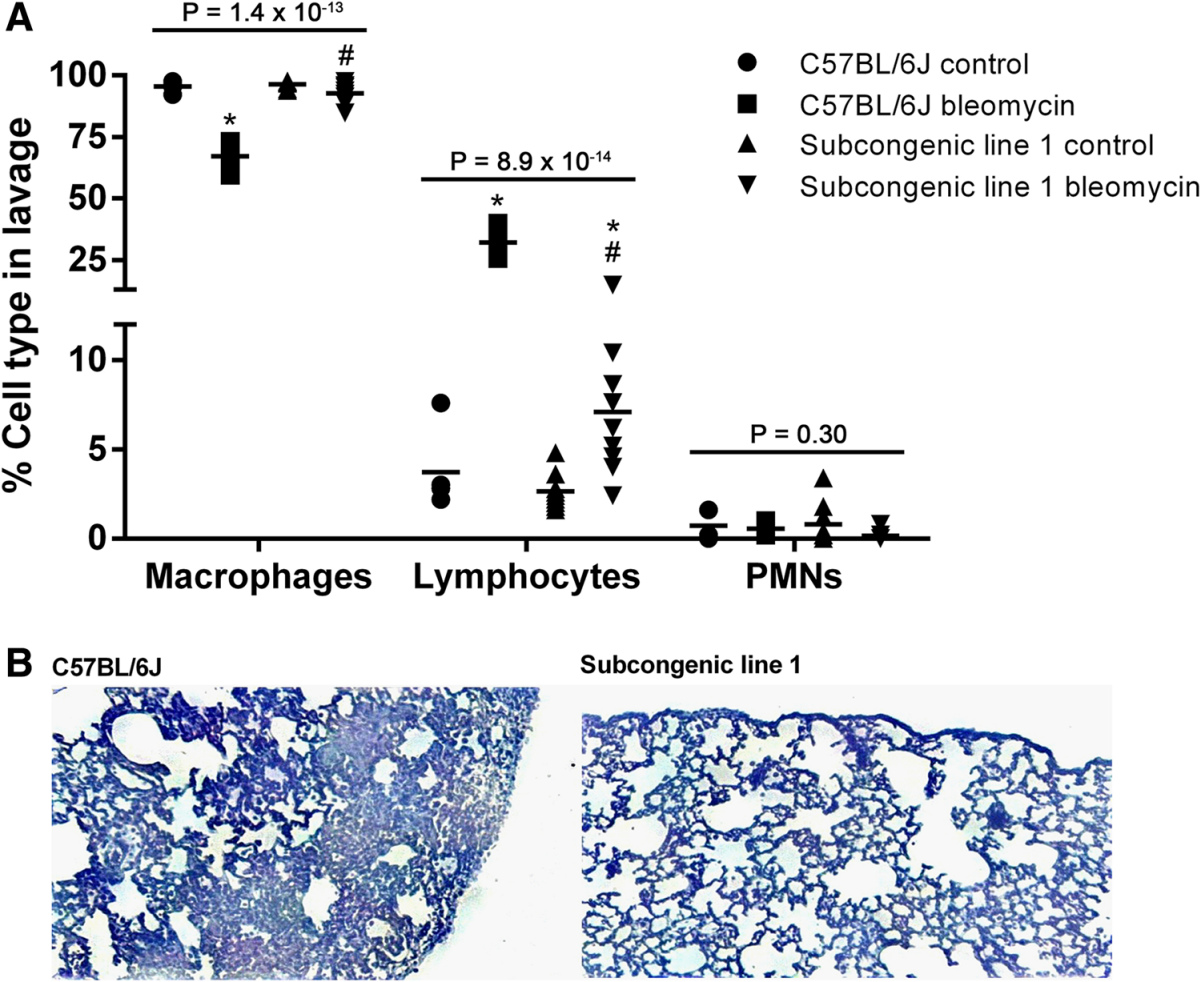
Bleomycin-induced pulmonary phenotype in C57BL/6J and subcongenic line 1 mice. Male mice were treated with bleomycin as in Fig. 1 and bronchoalveolar lavage and tissue samples were collected at necropsy. Cells in lavage samples were morphologically identified from cytospin preparations. **a** Cell percentages (mean ± SE) for groups of 5–10 mice. *P* values by ANOVA, Asterisk indicates a significant difference compared to corresponding control values, hash indicates a significant difference compared to C57BL/6J mice (*P* < 0.05), by Tukey’s post hoc test. **b** Images of Masson’s trichrome-stained lung sections indicating distinct fibrosis responses to bleomycin challenge; magnification ×50

The total cell number in the lavage of bleomycin-treated C57BL/6J mice was, on average of 3.56 ± 0.52 × 10^5^, and this number did not differ from the values for the subcongenic line 1 strain (3.04 ± 0.41 × 10^5^, *P* = 0.45). For each of C57BL/6J and subcongenic line 1 mice, the lavage cell count post bleomycin exceeded that of in strain untreated mice (*P* < 0.003) while cell counts in lavage of untreated mice did not differ between C57BL/6J and subcongenic line 1 mice (5.95 ± 2.04 × 10^4^, for C57BL/6J vs. the subcongenic line 1 strain 8.02 ± 2.59 × 10^4^, *P* = 0.54). Cell differential analyses revealed a significantly greater lymphocyte percentage in lavage of C57BL/6J mice, post bleomycin, than in the lavage of similarly treated subcongenic line 1 mice, as shown in Fig. 3, in support of a lymphocyte contribution to the fibrosis phenotype.

## Discussion

In summary, we have reduced the genomic region linked to susceptibility to a clear and clinically relevant pulmonary fibrosis phenotype to 0.71 Mb. Within this region, we identified 18 genes with DNA variants which could influence this trait and we show protection from fibrosis development to be associated with fewer lymphocytes in bronchoalveolar lavage.

Our strategy for reducing the *Blmpf1* genetic interval was to phenotype a panel of subcongenic mice and to evaluate the DNA variation within the identified set of positional candidate genes as has been used by others (Maegawa et al. 2018; Parkman et al. 2017). In detail, Parkman et al. (2017) studied lines of congenic mice to verify a locus of obesity and hypercholesterolemia, previously identified with linkage data, and interrogated the resultant gene list for variation with the potential to alter the protein sequence. Similarly, Maegawa et al. (2018) used DNA sequence comparisons to support a candidate gene for diabetes susceptibility in a genomic region identified through testing a panel of congenic mice. They, further, assessed whether the identified variant was associated with trait development in additional inbred strains, as we have here. Indeed, the bleomycin-induced lung disease of a panel of 23 inbred strains (Paun et al. 2013) and analyses with the mouseSNP map revealed significant associations to SNPs within five, of 11 evaluated, candidate genes of reduced *Blmpf1*. These data may be important supportive evidence for a candidate gene as contributing to the fibrosis trait, however, for this criterion, the inclusion of inbred strains other than C57BL/6J and C3H/HeJ is predicated on the assumption that the same loci, and the same genes, influence susceptibility to bleomycin-induced pulmonary disease in all evaluated strains. Since this is not known, a poor association of sequence variation and the inbred strain response was not used to exclude candidate genes.

There is support for variation within the reduced *Blmpf1* region to affect susceptibility to pulmonary fibrosis, clinically (Aquino-Galvez et al. 2009; Falfán-Valencia et al. 2005; Fingerlin et al. 2016; Libura et al. 2002). These findings include candidate gene studies of variant association to pulmonary fibrosis occurring as a consequence of bleomycin therapy (Libura et al. 2002), and of susceptibility to idiopathic pulmonary fibrosis (Aquino-Galvez et al. 2009; Falfán-Valencia et al. 2005). Most recently, Fingerlin et al. (2016) used a genome wide imputation strategy to identify a locus within the human leukocyte antigen (HLA) region to be associated with fibrotic interstitial pneumonia in 1616 patients. This locus includes the candidate gene *BTNL2*/*Btnl2* within one region of linkage disequilibrium.

Clinical (Daniil et al. 2005; DePianto et al. 2015; Feghali-Bostwick et al. 2007; Gross and Hunninghake 2001; Huang et al. 2015; Nuovo et al. 2012) and experimental (Kass et al. 2012; Wilson et al. 2010) data point to regulation of the adaptive immune response as an important contributor to pulmonary fibrosis development, and in this work, gene expression and inbred strain sequence evaluation revealed 18 candidate genes mapped to reduced *Blmpf1*, 9 of which function in immune regulation. Among these, candidate genes are four members of the butyrophilin-like (*Btnl*) family of receptors whose proteins are similar in structure to those of the B7 family (Arnett and Viney 2014) and function to modulate T cell responses (Rhodes et al. 2016). Butyrophilin-like genes of strain-dependent expression/deficiency (*Btnl1,2,4,6*) or protein sequence (*Btnl1,2,4,6*) may therefore affect bleomycin-induced injury susceptibility by altering the adaptive immune response of the lung. A second candidate gene which may influence strain-dependent immune responses to bleomycin challenge is *Notch 4* (Amsen et al. 2015), whose protein has been shown to augment an allergic airway inflammatory response in part by influencing T cell differentiation (Xia et al. 2018). In addition, the MHC class II gene *H2-Eb2* is also an immune-based candidate for fibrosis susceptibility owing to its function in antigen presentation (Monzón-Casanova et al. 2016). Finally, three genes of the complement component are within the 18 candidate genes of reduced *Blmpf1*. There is experimental evidence that reducing complement activation can decrease lung fibrosis (Cipolla et al. 2017), although our studies in the related model of radiation-induced pulmonary fibrosis revealed a *C4b* deficiency not to affect the fibrosis trait (Fox et al. 2013).

Additional candidate genes encode proteins which could affect bleomycin-induced pulmonary fibrosis through their DNA damage or epithelial cell functions. In detail, bleomycin treatment can lead to DNA damage (Bolzán and Bianchi 2018) thus variants in *Pbx2*, whose protein is DNA binding (Selleri et al. 2004), in *Stk19* encoding a nuclear locating kinase (Boeing et al. 2016) or in *Atf6b*, which encodes a transcription factor (Thuerauf et al. 2004) could create a strain-dependent response to bleomycin. There is also evidence that bleomycin damages epithelial cells in the pulmonary fibrosis process (Jin et al. 2018), thus genetic variants influencing epithelial cell function could affect susceptibility to fibrosis. For example, *Ager* encodes a surface protein expressed on the lung epithelium (Wolf et al. 2017), and candidate gene association studies have reported an increased risk (Yamaguchi et al. 2017) or no increased risk (Manichaikul et al. 2017) of the related trait of idiopathic pulmonary fibrosis in patients carrying an *AGER* minor allele. In addition, *Cyp21a1* encodes a hydroxylase expressed in the epithelium of the developing lung (Gilbert et al. 2017) and deficiencies in this hydroxylase result in congenital adrenal hyperplasia (El-Maouche et al. 2017), while the product of *Tnxb* is an extracellular matrix glycoprotein whose variants are associated with the connective tissue fragility of Ehlers–Danlos Syndrome (Chen et al. 2016; Mao et al. 2002). Neither gene has yet been linked to fibrosis.

In summary, our results reduced the genomic region of the major fibrosis susceptibility locus, *Blmpf1*, and pointed to genes known to regulate T cell numbers and activation (*Btnl* family, *H2-Eb2, Notch 4*), as among the limited list of potential causal variants leading to bleomycin-induced lung disease in mice. Studies in congenic mice supported this region as containing genetic variation which influences the lymphocyte response and pulmonary fibrosis.

## Electronic supplementary material

Below is the link to the electronic supplementary material.

**Supplemental Fig. 1**: Pulmonary expression of candidate genes in the reduced *Blmpf1* region. Data extracted from a gene expression by microarray study (Haston et al. 2005) wherein mice were treated as in Figure 1 or left untreated (controls). Relative gene expression in right lung tissue. * denotes a significant difference in expression between groups, *p* < 0.05. A. Genes with strain dependent expression in lungs of untreated control or bleomycin treated mice. B. Genes with bleomycin-induced expression in lungs of treated mice. C. Genes without strain or bleomycin dependent expression in lungs of untreated control or bleomycin treated mice. B6 = C57BL/6J (PPTX 51 KB)

## Data Availability

All data generated or analyzed during this study are included in this published article.
