# Supplementary material for: Fine mapping of the major bleomycin-induced pulmonary fibrosis susceptibility locus in mice
Source: Mamm Genome. 2018 Sep 1;29(9):670–9. doi: 10.1007/s00335-018-9774-3 (PMC6182746; doi:10.1007/s00335-018-9774-3)
Supplement: Supplementary file 1 — Supplemental Fig. 1: Pulmonary expression of candidate genes in the reduced Blmpf1 region. Data extracted from a gene expression by microarray study (Haston et al. 2005) wherein mice were treated as in Figure 1 or left untreated (controls). Relative gene expression in right lung tissue. * denotes a significant difference in expression between groups, p < 0.05. A. Genes with strain dependent expression in lungs of untreated control or bleomycin treated mice. B. Genes with bleomycin-induced expression in lungs of treated mice. C. Genes without strain or bleomycin dependent expression in lungs of untreated control or bleomycin treated mice. B6 = C57BL/6J (PPTX 51 KB) [file 335_2018_9774_MOESM1_ESM.pptx]

## Slide 1
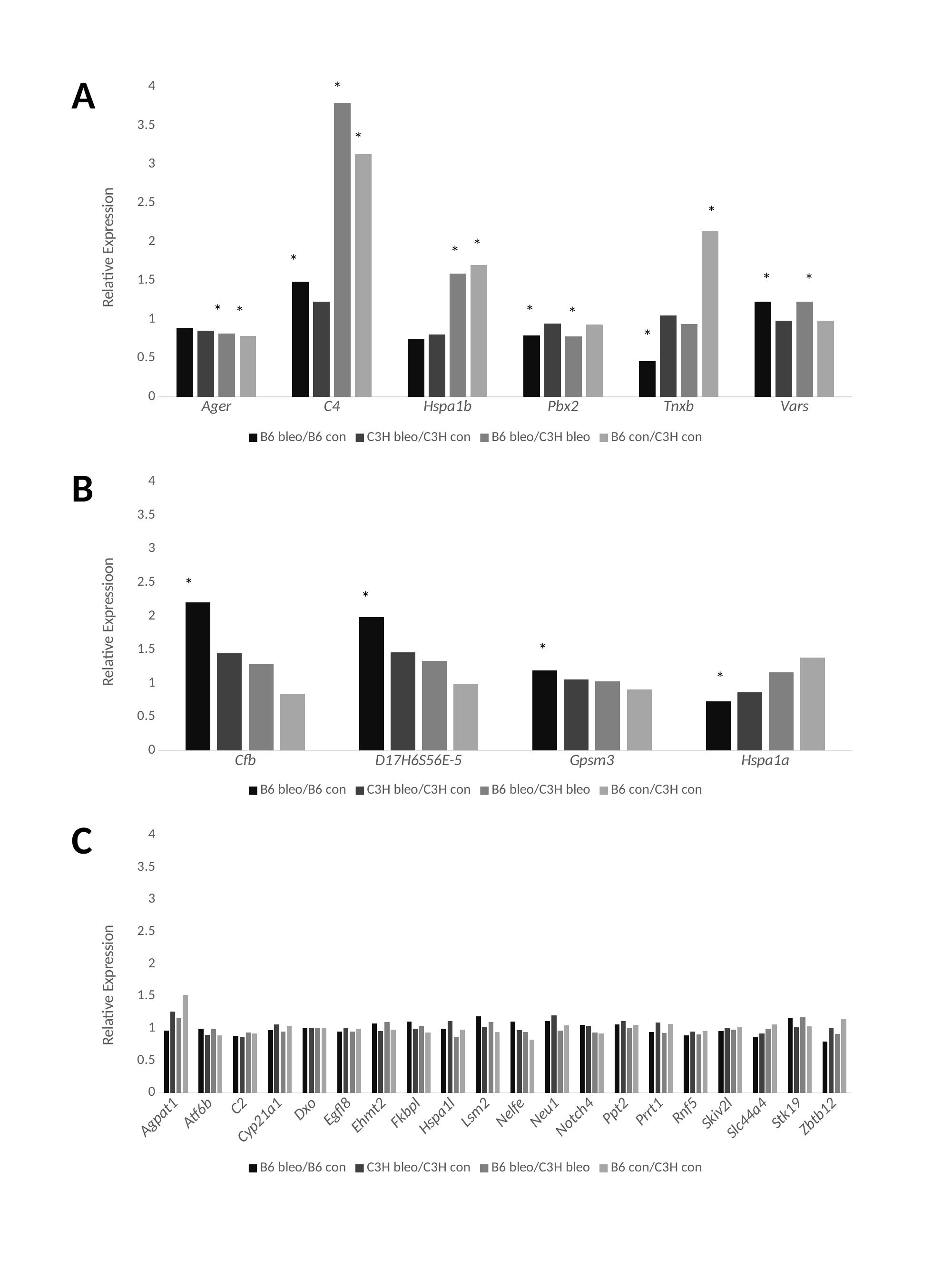

A
### Chart
| Category | B6 bleo/B6 con | C3H bleo/C3H con | B6 bleo/C3H bleo | B6 con/C3H con |
|---|---|---|---|---|
| Ager | 0.8920643902042731 | 0.8558230151154137 | 0.8155451267214524 | 0.7824124547260672 |
| C4 | 1.4864479476607315 | 1.225129209219202 | 3.798470169658321 | 3.1306960748405284 |
| Hspa1b | 0.7493929719946483 | 0.8050499996225274 | 1.5879951458917745 | 1.700353863893018 |
| Pbx2 | 0.7916507278219067 | 0.945312248417258 | 0.7814048699924949 | 0.9330776422187248 |
| Tnxb | 0.45960872375928247 | 1.0477970309005737 | 0.938882574271253 | 2.1404258074982536 |
| Vars | 1.2294074252291787 | 0.9814956007692993 | 1.2264719588755049 | 0.9791520755446963 |*
*
*
*
*
*
*
*
*
*
*
*
*
B
### Chart
| Category | B6 bleo/B6 con | C3H bleo/C3H con | B6 bleo/C3H bleo | B6 con/C3H con |
|---|---|---|---|---|
| Cfb | 2.2031899930264323 | 1.4444130603694005 | 1.2922717477640737 | 0.8472143555140156 |
| D17H6S56E-5 | 1.9849955836651403 | 1.462450968767673 | 1.3376267118275498 | 0.9855001676878898 |
| Gpsm3 | 1.1925076252659181 | 1.0566009934782017 | 1.0266666189325642 | 0.9096604051426143 |
| Hspa1a | 0.729727380642002 | 0.8673180022998436 | 1.1661293622916873 | 1.3860038909821295 |*
*
*
*
C
### Chart
| Category | B6 bleo/B6 con | C3H bleo/C3H con | B6 bleo/C3H bleo | B6 con/C3H con |
|---|---|---|---|---|
| Agpat1 | 0.9628954237689106 | 1.259830301781897 | 1.164121124968985 | 1.5231093969061487 |
| Atf6b | 0.9922843277815455 | 0.9014522203690558 | 0.984860489010863 | 0.8947079478292937 |
| C2 | 0.8816447632058262 | 0.8626030161809671 | 0.9397770066763408 | 0.9194797205496219 |
| Cyp21a1 | 0.9701455650180209 | 1.0650058112800842 | 0.9480650927933116 | 1.0407663238432836 |
| Dxo | 1.0015310521781629 | 1.0054495219946107 | 1.0086008904644153 | 1.0125470208789766 |
| Egfl8 | 0.9507879558759588 | 1.000945312693034 | 0.9487345842440068 | 0.9987836185976083 |
| Ehmt2 | 1.0763105655516887 | 0.9558539848468797 | 1.1023150045196803 | 0.9789481060111744 |
| Fkbpl | 1.1026379205067025 | 0.9940594012330748 | 1.0367490864604347 | 0.9346587460389535 |
| Hspa1l | 0.9930256388815275 | 1.114983580258906 | 0.8710883555558104 | 0.9780706311806862 |
| Lsm2 | 1.1866163068473714 | 1.016744075629105 | 1.097290871689783 | 0.9402061867804626 |
| Nelfe | 1.1094410121159757 | 0.9731805068171782 | 0.943596313474887 | 0.8277046986273833 |
| Neu1 | 1.114538965571971 | 1.2056614032293107 | 0.9680958165375618 | 1.0472453603523026 |
| Notch4 | 1.0558346759781856 | 1.0372945491131333 | 0.9388280361303911 | 0.9223425092857609 |
| Ppt2 | 1.0590798892044893 | 1.1120066109772544 | 1.0041003250769043 | 1.0542794844387222 |
| Prrt1 | 0.9467214762067986 | 1.0922381843141422 | 0.9282389620809172 | 1.0709147981041882 |
| Rnf5 | 0.8948977207398648 | 0.9481069063974311 | 0.9043982146093534 | 0.9581722844211898 |
| Skiv2l | 0.9592705461238714 | 0.9991630102654685 | 0.9807780329442036 | 1.021564912795969 |
| Slc44a4 | 0.8657561441753795 | 0.9218439618977383 | 0.998905697260298 | 1.0636195789309002 |
| Stk19 | 1.1569406260243642 | 1.0148179633612109 | 1.1758957971519663 | 1.0314446144841631 |
| Zbtb12 | 0.795666616489945 | 0.9993979428916773 | 0.9162352878100003 | 1.1508383572526693 |
